# Indoxyl Sulfate and Its Potential Role in Mineralocorticoid Receptor Transactivation in Chronic Kidney Disease

**DOI:** 10.7759/cureus.75236

**Published:** 2024-12-06

**Authors:** Akiko Kudo, Akihiro Fukuda, Koro Gotoh, Hirotaka Shibata

**Affiliations:** 1 Department of Endocrinology, Metabolism, Rheumatology and Nephrology, Faculty of Medicine, Oita University, Yufu, JPN; 2 Faculty of Welfare and Health Science, Oita University, Oita, JPN

**Keywords:** aldosterone, chronic kidney disease (ckd), indoxyl sulfate, mineralocorticoid receptor, uremic toxins

## Abstract

Background: The uremic toxin indoxyl sulfate (IS) is an important factor in chronic kidney disease (CKD) progression. Inhibitors of the renin-angiotensin system and add-on therapy with mineralocorticoid receptor (MR) antagonists can help reduce proteinuria and suppress CKD progression. However, the association between IS and MR activation remains unknown.

Materials and Methods: *In vivo* experiments utilized the 5/6 nephrectomy model to assess mineralocorticoid receptor (MR) activation in chronic kidney disease (CKD). The clinical parameters and immunohistochemical analysis of IS and MR proteins were investigated. *In vitro* experiments involved transfecting COS-7 cells with MR expression plasmids and MR response element-luciferase reporter plasmids. The cells were then treated with aldosterone (10⁻¹⁰ mol/L), indoxyl sulfate (IS, 500 μmol/L), and α-lipoic acid (10⁻³ mol/L). MR transcriptional activity was investigated by luciferase assays, and protein levels were measured by Western blotting.

Results: In the 5/6 nephrectomy model, the serum IS concentration was significantly increased; however, the plasma aldosterone levels were decreased. Immunohistochemistry showed that the expression of IS protein increased in injured tubular cells in the 5/6 nephrectomy group compared with that in the sham group. Furthermore, evaluations of serial kidney sections revealed that the expression site of IS protein was colocalized with the distal nephron, where the expression of MR protein was observed. MR-mediated transcriptional activity in COS-7 cells was increased in an aldosterone concentration-dependent manner. IS increased MR-mediated transcriptional activity and protein levels with and without aldosterone, and α-lipoic acid attenuated this increase.

Conclusions: IS could enhance MR transactivation by increasing MR protein levels through oxidative stress in CKD rats, indicating that treatment with MR antagonists and antioxidants may play a permissive role in inhibiting IS-induced CKD progression.

## Introduction

The number of patients with chronic kidney disease (CKD) is increasing worldwide [1]. CKD is not only the leading cause of end-stage kidney disease but also a significant risk factor for cardiovascular events and mortality. CKD progression has multiple pathogenesis, including aging, diabetes, hypertension, glomerulonephritis, and ambient heat stress [2]. The mechanisms of nephropathy progression are not fully understood, and strategies for CKD vary depending on the underlying diseases. However, uremic toxins commonly accumulate in the body because of renal dysfunction in any cause of CKD. Previous studies have reported that indoxyl sulfate (IS), a major uremic toxin, leads to CKD by inducing tubular and podocyte injury, vascular inflammation, and calcification [3-5]. Furthermore, IS also causes systemic organ damage, such as cardiac inflammation and fibrosis, leading to atrial fibrillation [6]. These previous studies have suggested that IS removal is an important factor in inhibiting CKD progression.

Recent large-scale clinical trials have demonstrated that sodium-glucose cotransporter-2 inhibitors and MR antagonists, in addition to renin-angiotensin system inhibitors, are useful in both diabetic and non-diabetic CKD [7,8], and the use of MR antagonists to suppress CKD progression has attracted attention. MR activation is caused by aldosterone or cortisol as a ligand; however, MR activation is possibly not mediated by aldosterone but by abnormalities in the transcription factor regulatory system. We have proposed that “MR-associated hypertension and its organ damage” exists in patients with diabetes, obesity, and CKD, in which MR signaling is enhanced regardless of an increase in plasma aldosterone levels [9]. Various mechanisms have been proposed for kidney injury associated with excessive MR activation, including podocyte damage and fibrosis of the interstitium, and kidney injury can be suppressed by MR antagonists [10]. However, the mechanism of MR activation-mediated nephropathy progression is still unclear, and its clarification may lead to new therapies to control the progression of nephropathy.

Despite reports on the relationship between IS and renin-angiotensin-aldosterone system (RAAS) activation, including increased renin expression in cultured rat mesangial cells [11] and the finding that IS activates renal RAAS and induces renal fibrosis [12], no studies have reported the relationship between IS and MR activation. Thus, this study aimed to clarify the relationship between IS and MR activation in CKD and to develop the treatment of CKD.

The content of this article was previously presented as a meeting poster at the annual meeting of the Japanese Society of Nephrology on August 19, 2020.

## Materials and methods

Animal experiments

The animal protocols were approved by the institutional animal care and use committee of Oita University (Approval number: 181503). Eight-week-old male Sprague-Dawley rats (KYODO Co. Ltd., Japan) underwent a staged nephrectomy, starting with a 2/3 resection of the left kidney, followed by a right nephrectomy seven days later, completing a 5/6 nephrectomy procedure (n=6) [6]. The control group underwent a sham procedure without nephrectomy (n=6). The surgeries were performed with intraperitoneal anesthetic administration. Both groups were anesthetized by intraperitoneal injection, and blood samples were collected and euthanized at 10 weeks (aged 19 weeks) after 5/6 nephrectomy. 24-hour urine collection was performed using metabolic cages at 19 weeks. Urinary protein, serum creatinine, and plasma aldosterone (chemiluminescent enzyme immunoassay) were measured by SRL, Inc. (Tokyo, Japan). Serum IS levels were measured by FUSHIMI Pharmaceutical Clinical Laboratory Center (Marukame, Japan). Kidney tissues were removed during euthanization.

Cell culture, transfections, and luciferase assays

COS-7 cells were provided by the RIKEN BRC through the National Bio-Resource Project of the MEXT/AMED, Japan. COS-7 cells were transfected, and luciferase assays were performed as described previously [13,14]. COS-7 cells were routinely maintained in Dulbecco’s modified eagle medium (DMEM, FUJIFILM, Tokyo, Japan) supplemented with 10% fetal bovine serum (Funakoshi Co. Ltd. Tokyo, Japan) and 1% penicillin/streptomycin (FUJIFILM). At 24 hours before transfection, 1×10^5^ cells per well of a 24-well dish were plated in Opti-MEM (Thermo Fisher Scientific, Tokyo, Japan). All transfections were performed using lipofectamine 2000 (Invitrogen, MA, USA) with 0.3 μg/well of the luciferase reporter, 0.01 μg/well of pRL-null (Promega, WI, USA) internal control plasmids, and the indicated amounts of expression plasmids according to the manufacturer’s instructions. After 24 hours, the medium was changed to DMEM with IS (Cayman Chemical Company, MI, USA), aldosterone (Sigma-Aldrich, MO, USA) or α-lipoic acid (FUJIFILM). After an additional 24-48 hours, the cell extracts were assayed for both Firefly and Renilla luciferase activities with a dual-luciferase reporter assay system (Promega). Relative luciferase activity was determined as the ratio of Firefly/Renilla luciferase activities, and data were expressed as the mean (±S.E.) of triplicate values obtained from a representative experiment that was independently repeated at least three times.

Plasmid construct

All experimental protocols using recombinant DNA were approved by the Oita University Genetic Modification Safety Committee (Approval number: 29-8, 3-20, 3-33). 3×MRE-E1b-Luc and pcDNA3.1-hMR(1-984) were gifts from Dr. Yokota (St. Marianna University School of Medicine).

Western blot analysis

The cells were lysed with RIPA buffer (Cell Signaling Technology, MA, USA) including phenylmethanesulfonyl fluoride (Cell Signaling Technology). Protein concentrations were measured using the Bradford method [15]. Proteins were then separated on 7.5% polyacrylamide gels (Bio-Rad, CA, USA) and transferred onto nitrocellulose membranes (Bio-Rad). The primary antibodies used for the Western blots were anti-MR (PP-H2133-00, Perseus Proteomics Inc., Tokyo, Japan) or anti-GAPDH (Sigma-Aldrich) antibodies.

Immunohistochemistry

Kidney tissues cut into two μm sections were fixed in formalin and embedded in paraffin. Immunostaining of IS and MR in renal sections was performed using the streptavidin-biotinylated peroxidase complex method. The primary antibodies used for anti-MR antibodies were gifts from Dr. C. Gomez-Sanchez [16] or anti-IS antibodies (Trans Genic Inc., Kobe, Japan) and incubated overnight at 4℃. Biotin-conjugated secondary anti-mouse antibody (Vector Laboratories, CA, USA) was applied for two hours and visualized using a 3,3′-diaminobenzidine substrate kit (Vector Laboratories) as immunostaining-positive areas and counterstained with hematoxylin.

Statistical analysis

 A t-test or one-way analysis of variance (ANOVA) was utilized for statistical analysis between the two groups. The variance of the intended two groups was assayed by the F test in advance, and a corresponding t-test was then performed. Two-way ANOVA was utilized for statistical comparisons among more than three groups. For the multiple comparisons, Tukey’s honestly significant difference was utilized as the post hoc analysis. All data are expressed as mean±S.E. p<0.05 was considered significant.

## Results

MR activation and IS accumulation in the 5/6 nephrectomy rat model

**Figure 1:**
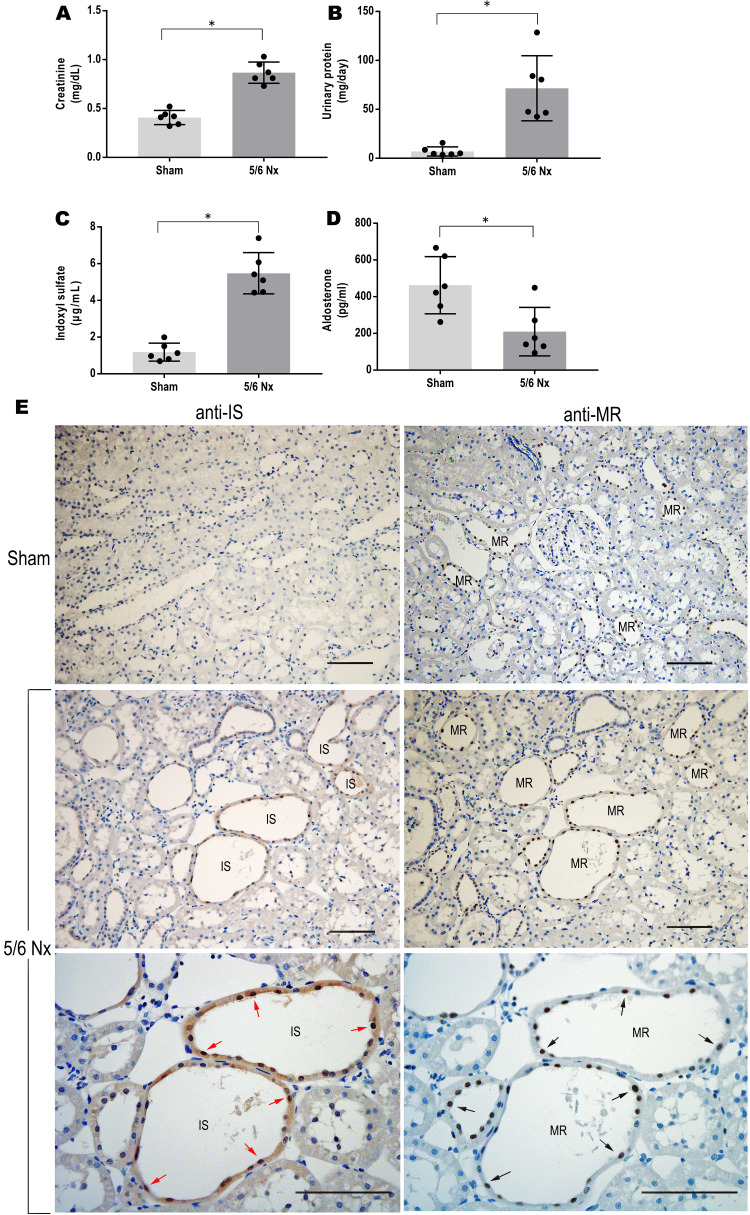
Clinical parameters and immunohistological findings in the sham and 5/6 nephrectomy rat models. A–C: serum levels of creatinine, indoxyl sulfate (IS), and urinary proteins were significantly increased in the 5/6 nephrectomy (5/6 Nx) group compared with the sham group, n=6 each group, *p<0.01; D: plasma levels of aldosterone decreased in the 5/6 Nx group, *p<0.05 vs. sham group; E: Immunohistochemical findings with anti-mineralocorticoid receptor (MR) antibody or anti-IS antibody. MR proteins were expressed in the distal/collecting ducts in both sham and 5/6 Nx groups. IS accumulation was observed in the damaged tubules in the 5/6 Nx rat model. IS expression (red arrow) was colocalized in the distal/collecting ducts where MR proteins were expressed (black arrow) by serial section evaluation; Scale bar: 100 μm.

First, a 5/6 nephrectomy (5/6 Nx) model was created to see if increased IS and MR activation could be observed in the CKD model. The 5/6 Nx group showed significantly high levels of serum creatinine, IS, and urinary proteins compared with the sham group but decreased plasma aldosterone levels (Figure 1A-1D). The plasma active renin concentration did not differ between the two groups (<0.2 pg/mL). Immunostaining showed sporadic MR protein expression in the distal/collecting ducts in both the sham and 5/6 Nx groups (Figure 1E). In addition, the 5/6 Nx group increased IS expression in the proximal, distal, and especially of injured tubules compared with the sham group, while the glomeruli were hardly stained. Furthermore, evaluation of serial kidney sections revealed that the expression site of the IS protein was colocalized with the distal nephrons with MR protein expression (Figure 1E).

Enhancement of the aldosterone-mediated MR transcriptional activity by IS in COS-7 cells

**Figure 2:**
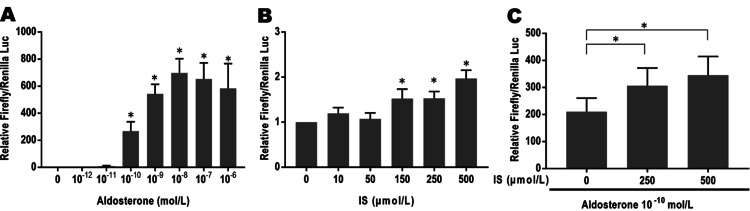
Aldosterone and indoxyl sulfate enhanced the mineralocorticoid receptor transcriptional activity in COS-7 cells. A: concentration-response curve to aldosterone. COS-7 cells were transfected with MR reporter, incubated with or without aldosterone (10^−12^ to 10^−6^ mol/L) for 24 hours, and luciferase reporter assay was performed. n=4 for each group. *p<0.01 vs. aldosterone 0. B: effects of IS (10-500 μmol/L) on luciferase activity without aldosterone. n=4 for each group. *p<0.01 vs. IS 0. C: effects of IS (250–500 μmol/L) on luciferase activity with aldosterone 10^−10^ mol/L, n=5 each group. *p<0.01 vs. IS 0.

Then, to examine the relationship between IS and MR activation in vitro, plasmids encoding MR and mineralocorticoid response element-luciferase were transfected into COS-7 cells and evaluated by a luciferase reporter assay. Treatment with aldosterone (10^−10^ to 10^−6^ mol/L) increased the MR-driven luciferase reporter activity in a concentration-dependent manner (Figure 2A). Treatment with IS (10-500 μmol/L) increased MR-driven luciferase activity without ligand stimulation, and IS concentrations >150 μmol/L significantly enhanced MR transcriptional activity (Figure 2B). Furthermore, the addition of IS to aldosterone-treated COS-7 cells further enhanced MR-mediated luciferase activity (Figure 2C). These findings indicated that IS may, in part, enhance MR transactivation with and without aldosterone.

 Antioxidants inhibit MR activation by IS administration

**Figure 3:**
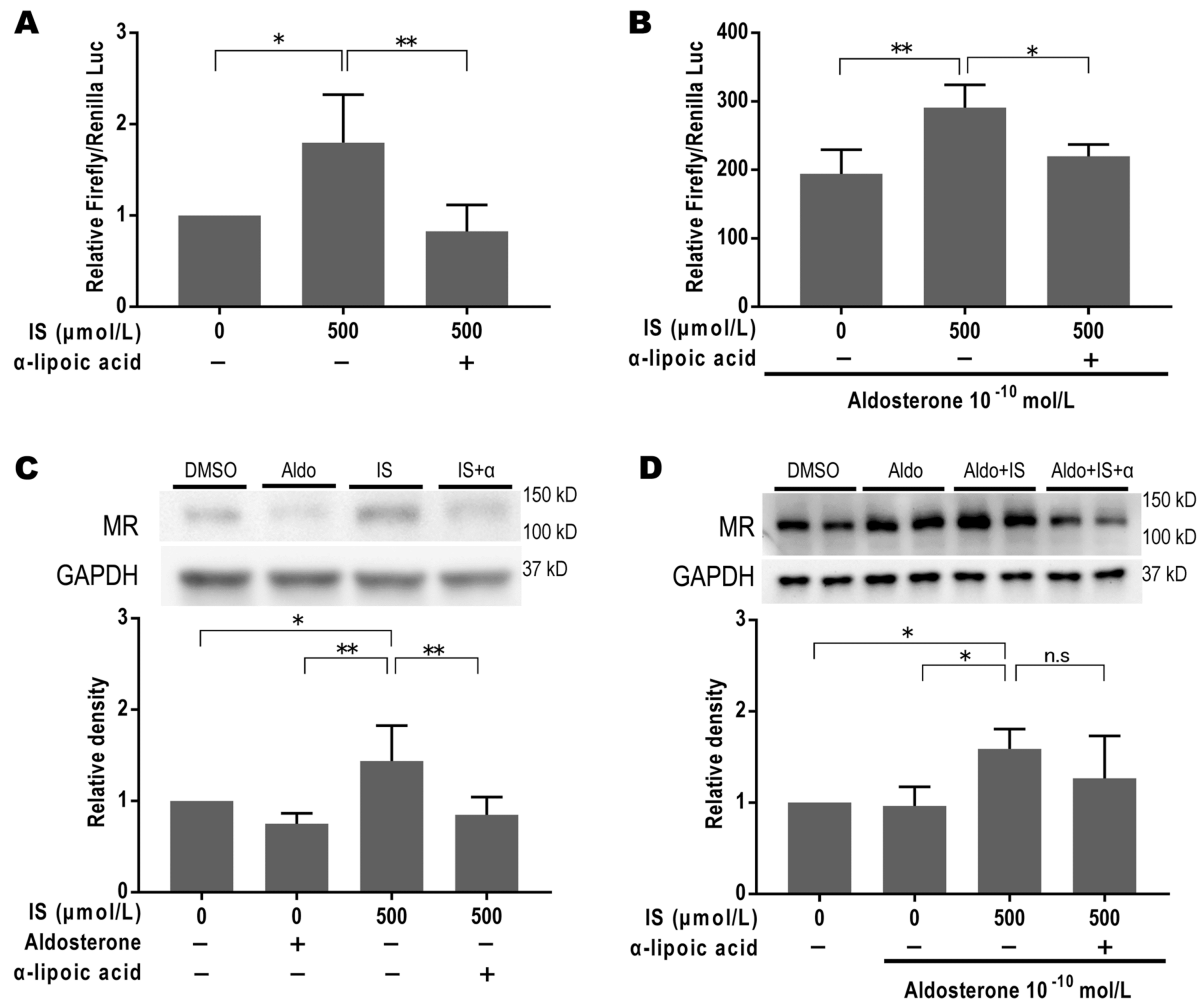
Antioxidants inhibited mineralocorticoid receptor activation by indoxyl sulfate in COS-7 cells. A, B : luciferase reporter assay. Mineralocorticoid receptor (MR) transcriptional activity by indoxyl sulfate (IS) was significantly suppressed by α-lipoic acid (10−3 mol/L) without (A) and with (B) aldosterone, n=5 each group, *p<0.05, **p<0.01; C: the increase in MR protein levels by IS was significantly suppressed by α-lipoic acid without aldosterone, n=6 each group, *p<0.05, **p< 0.01; D: with aldosterone, IS significantly increased MR protein expression; however, the suppression of MR protein levels by α-lipoic acid was not significant, n=5 each group, *p<0.05, DMSO: dimethyl sulfoxide.

IS causes renal tubular cell damage by producing reactive oxygen species and disrupting the antioxidant system. Whether MR activation by IS could be suppressed by antioxidants was investigated. MR transcriptional activity (Figure 3A, 3B) and protein levels (Figure 3C) enhanced by IS (500 μmol/L) were significantly suppressed by α-lipoic acid (10^−3^ mol/L). A similar effect was observed in the presence of aldosterone (10^−10^ mol/L), although the inhibitory effect of α-lipoic acid at the MR protein level was not significant (Figure 3D). These results indicated that the enhancement of MR transactivation by IS is partly due to the elevation of MR protein levels.

## Discussion

This study showed that IS is partially responsible for MR activation in CKD, and α-lipoic acid, an antioxidant agent, inhibited IS-induced potentiation of MR transactivation.

This study focused on three points. The first point is how IS potentiates MR transactivation in the CKD rat model. MR activation is triggered by aldosterone or cortisol as a ligand; however, ligand-independent MR activation mechanisms such as increased transcription and sensitivity, stabilization, and other factors are also considered [13,17-19]. The pathological conditions of ligand-independent MR activation include salt-sensitive hypertension, diabetes, CKD, dyslipidemia, and metabolic syndrome. These conditions often present with treatment-resistant hypertension, and add-on therapy with MR antagonists improves blood pressure and organ damage, even with normal plasma aldosterone levels [20,21]. We have proposed such clinical pathogenesis as MR-associated hypertension and its organ damage, in which MR is pathologically activated regardless of plasma aldosterone levels [9]. The 5/6 Nx group in this study showed decreased plasma aldosterone levels compared with the sham group. In the 5/6 Nx group, the renin-angiotensin-aldosterone system may have been suppressed due to body fluid retention. In the sham group, the renin concentration may have been decreased at the time of blood collection due to several factors, such as anesthetics, but aldosterone was not affected. Therefore, the 5/6 Nx group may have induced MR-associated renal damage regardless of plasma aldosterone levels. Previously, we reported that under hyperglycemic conditions in diabetic nephropathy, de-ubiquitination of MR protein is induced by phosphorylation by the protein kinase C pathway [14] and *O*-linked N-acetylglucosamine (*O*-GlcNAc) modification by the hexosamine pathway, resulting in the elevation of MR protein levels and aldosterone-mediated MR transactivation [22].

On the other hand, uremic toxins containing IS increase oxidative stress and the production of reactive oxygen species [23], followed by renal tubular cell damage and progression of cardiovascular disease and bone disease in patients with CKD [24,25]. Oxidative stress induced glomerular MR activation through increased MR gene transcription [26], whereas MR antagonists improved endothelial dysfunction by enhancing the bioavailability of nitric oxide and decreasing superoxide anion levels [27]. Thus, oxidative stress is not only a cause of MR transactivation but also a target for CKD treatment. In this study, we found that IS-induced MR activation is due, at least in part, to the elevation of MR protein levels. Immunohistochemical analysis showed that MR proteins were expressed in the nuclei of the distal/collecting ducts and colocalized with the injured tubules in which IS was accumulated in the CKD rat model, suggesting that the sites of tubular damage due to MR activation were consistent with those of IS. In addition, IS was found to potentiate aldosterone-mediated MR transactivation, the activity of which was suppressed by α-lipoic acid in COS-7 cells. α-lipoic acid has various biological actions such as energy modulator and redox modulator, among them great antioxidant potential [28]. Based on these data, oxidative stress by IS may play a role in enhancing MR transactivation through the post-translational modification of MR proteins, followed by decreased ubiquitination, thus resulting in the elevation of MR protein levels. MR luciferase activities in the absence of aldosterone were very low, suggesting that MR transactivation by IS may occur mainly in the presence of aldosterone. The detailed molecular mechanisms for MR proteins remain to be elucidated in future studies.

Second, determining which cells in the kidney are affected by IS and MR activation and what they do there would be crucial. A previous study reported that IS is mainly localized in the renal proximal tubular cells, particularly in dilated tubules in uremic rats, and exacerbates tubulointerstitial injuries [29]. In contrast, MR expression has been observed in the distal nephron, such as the distal tubules and cortical collecting duct cells. In this study, IS accumulated in the distal tubules where MR proteins were expressed, indicating that IS may affect MR activation in some distal tubules. A recent study showed that aldosterone activates transporters and pumps such as Na+ and K+-ATPase through MR transactivation in the proximal tubules in rat kidneys [30]. We have found high expression of *O*-GlcNAc transferase in the proximal tubular cells of the kidney, and the increase in *O*-GlcNAc modification under hyperglycemic conditions may lead to MR overactivation in the proximal tubular cells [31]. Various studies have reported on which site of the tubule MR activation is triggered; however, further studies are required. Renal tubular cells with IS-related injury release transforming growth factor-β or other chemokines such as intercellular adhesion molecule-1, monocyte chemoattractant protein-1, osteopontin, and endothelin-1 [32], which promote renal fibrosis. Some of these chemokines were increased in response to MR expression levels in renal biopsies of patients with proteinuria [17]. These proinflammatory factors or profibrotic factors may be responsible for the damage in tubular cells where IS accumulation and MR expression are colocalized.

Third, we discuss the clinical significance of this study. Recent large clinical trials of the effects of finerenone, a novel MR antagonist, on chronic kidney disease with type 2 diabetes have shown a significant reduction in cardiovascular and renal composite outcomes [33,34]. Conversely, AST-120, an oral carbon adsorbent that lowers IS levels by reducing the absorption of indole, improved the rate of the estimated glomerular filtrate rate decline in the CAP-KD study [35]. However, the latest recent large clinical trials (EPPIC trials) have shown that AST-120 treatment was not significantly effective in reducing renal events compared with the placebo, proposing that the reduction of IS accumulation with AST-120 is insufficient to prevent CKD progression [36,37]. The results of the present study and recent clinical trials indicate that IS may play a permissive role in the pathogenesis of CKD progression partly by enhancing MR overactivation by the elevation of MR protein levels.

This study has several limitations. First, the detailed molecular mechanisms of how IS increases MR protein levels remain to be elucidated. Second, the role of IS in other CKD models, such as diabetes and hypertension, also remains to be investigated. Third, it is necessary to know the effect of IS-mediated MR transactivation on albuminuria/proteinuria and CKD progression in vivo. Further studies are needed to clarify these issues.

## Conclusions

Indoxyl sulfate could contribute to kidney damage partly by enhancing MR activation in 5/6 nephrectomy CKD rats. Oxidative stress by IS may be responsible for MR transactivation through MR protein levels elevation in CKD. Treatment with MR antagonists and antioxidants may play a permissive role in inhibiting IS-induced CKD progression.

## References

[1] Bikbov B, Purcell C, Levey AS (2020). Global, regional, and national burden of chronic kidney disease, 1990-2017: a systematic analysis for the Global Burden of Disease Study 2017. Lancet.

[2] Zhang Z, Heerspink HJL, Chertow GM (2024). Ambient heat exposure and kidney function in patients with chronic kidney disease: a post-hoc analysis of the DAPA-CKD trial. Lancet Planet Health.

[3] Ichii O, Otsuka-Kanazawa S, Nakamura T (2014). Podocyte injury caused by indoxyl sulfate, a uremic toxin and aryl-hydrocarbon receptor ligand. PLoS One.

[4] Chen J, Zhang X, Zhang H (2016). Indoxyl sulfate enhance the hypermethylation of klotho and promote the process of vascular calcification in chronic kidney disease. Int J Biol Sci.

[5] Ito S, Osaka M, Edamatsu T, Itoh Y, Yoshida M (2016). Crucial role of the aryl hydrocarbon receptor (AhR) in indoxyl sulfate-induced vascular inflammation. J Atheroscler Thromb.

[6] Aoki K, Teshima Y, Kondo H (2015). Role of indoxyl sulfate as a predisposing factor for atrial fibrillation in renal dysfunction. J Am Heart Assoc.

[7] Heerspink HJ, Stefánsson BV, Correa-Rotter R (2020). Dapagliflozin in patients with chronic kidney disease. N Engl J Med.

[8] Provenzano M, Puchades MJ, Garofalo C (2022). Albuminuria-lowering effect of dapagliflozin, eplerenone, and their combination in patients with chronic kidney disease: A randomized cross-over clinical trial. J Am Soc Nephrol.

[9] Shibata H, Itoh H (2012). Mineralocorticoid receptor-associated hypertension and its organ damage: clinical relevance for resistant hypertension. Am J Hypertens.

[10] Fujihara CK, Kowala MC, Breyer MD (2017). A novel aldosterone antagonist limits renal injury in 5/6 nephrectomy. Sci Rep.

[11] Wang WJ, Cheng MH, Sun MF, Hsu SF, Weng CS (2014). Indoxyl sulfate induces renin release and apoptosis of kidney mesangial cells. J Toxicol Sci.

[12] Sun CY, Chang SC, Wu MS (2012). Uremic toxins induce kidney fibrosis by activating intrarenal renin-angiotensin-aldosterone system associated epithelial-to-mesenchymal transition. PLoS One.

[13] Yokota K, Shibata H, Kurihara I (2007). Coactivation of the N-terminal transactivation of mineralocorticoid receptor by Ubc9. J Biol Chem.

[14] Hayashi T, Shibata H, Kurihara I (2017). High glucose stimulates mineralocorticoid receptor transcriptional activity through the protein kinase C β signaling. Int Heart J.

[15] Kume O, Takahashi N, Wakisaka O (2011). Pioglitazone attenuates inflammatory atrial fibrosis and vulnerability to atrial fibrillation induced by pressure overload in rats. Heart Rhythm.

[16] Gomez-Sanchez CE, de Rodriguez AF, Romero DG, Estess J, Warden MP, Gomez-Sanchez MT, Gomez-Sanchez EP (2006). Development of a panel of monoclonal antibodies against the mineralocorticoid receptor. Endocrinology.

[17] Quinkler M, Zehnder D, Eardley KS (2005). Increased expression of mineralocorticoid effector mechanisms in kidney biopsies of patients with heavy proteinuria. Circulation.

[18] Mitsuishi Y, Shibata H, Kurihara I (2018). Epidermal growth factor receptor/extracellular signal-regulated kinase pathway enhances mineralocorticoid receptor transcriptional activity through protein stabilization. Mol Cell Endocrinol.

[19] Shibata S, Nagase M, Yoshida S (2008). Modification of mineralocorticoid receptor function by Rac1 GTPase: implication in proteinuric kidney disease. Nat Med.

[20] Williams B, MacDonald TM, Morant S (2015). Spironolactone versus placebo, bisoprolol, and doxazosin to determine the optimal treatment for drug-resistant hypertension (PATHWAY-2): a randomised, double-blind, crossover trial. Lancet.

[21] Mukoyama M (2024). Treatment with a mineralocorticoid receptor blocker esaxerenone on top of the first-line therapy: promise in uncontrolled hypertension. Hypertens Res.

[22] Jo R, Shibata H, Kurihara I (2023). Mechanisms of mineralocorticoid receptor-associated hypertension in diabetes mellitus: the role of O-GlcNAc modification. Hypertens Res.

[23] Tanaka S, Watanabe H, Nakano T (2020). Indoxyl sulfate contributes to adipose tissue inflammation through the activation of NADPH oxidase. Toxins (Basel).

[24] Li Q, Zhang S, Wu QJ (2022). Serum total indoxyl sulfate levels and all-cause and cardiovascular mortality in maintenance hemodialysis patients: a prospective cohort study. BMC Nephrol.

[25] Fujii H, Goto S, Fukagawa M (2018). Role of uremic toxins for kidney, cardiovascular, and bone dysfunction. Toxins (Basel).

[26] Kitada K, Nakano D, Liu Y (2012). Oxidative stress-induced glomerular mineralocorticoid receptor activation limits the benefit of salt reduction in Dahl salt-sensitive rats. PLoS One.

[27] González-Blázquez R, Somoza B, Gil-Ortega M (2018). Finerenone attenuates endothelial dysfunction and albuminuria in a chronic kidney disease model by a reduction in oxidative stress. Front Pharmacol.

[28] Kamt SF, Liu J, Yan LJ (2023). Renal-protective roles of lipoic acid in kidney disease. Nutrients.

[29] Miyazaki T, Aoyama I, Ise M, Seo H, Niwa T (2000). An oral sorbent reduces overload of indoxyl sulphate and gene expression of TGF-beta1 in uraemic rat kidneys. Nephrol Dial Transplant.

[30] Salyer SA, Parks J, Barati MT, Lederer ED, Clark BJ, Klein JD, Khundmiri SJ (2013). Aldosterone regulates Na(+), K(+) ATPase activity in human renal proximal tubule cells through mineralocorticoid receptor. Biochim Biophys Acta.

[31] Jo R, Itoh H, Shibata H (2024). Mineralocorticoid receptor overactivation in diabetes mellitus: role of O-GlcNAc modification. Hypertens Res.

[32] Liu WC, Tomino Y, Lu KC (2018). Impacts of indoxyl sulfate and p-Cresol sulfate on chronic kidney disease and mitigating effects of AST-120. Toxins (Basel).

[33] Bakris GL, Agarwal R, Anker SD (2020). Effect of finerenone on chronic kidney disease outcomes in type 2 diabetes. N Engl J Med.

[34] Pitt B, Filippatos G, Agarwal R (2021). Cardiovascular events with finerenone in kidney disease and type 2 diabetes. N Engl J Med.

[35] Akizawa T, Asano Y, Morita S (2009). Effect of a carbonaceous oral adsorbent on the progression of CKD: a multicenter, randomized, controlled trial. Am J Kidney Dis.

[36] Schulman G, Berl T, Beck GJ (2015). Randomized placebo-controlled EPPIC trials of ast-120 in CKD. J Am Soc Nephrol.

[37] Schulman G, Berl T, Beck GJ (2016). The effects of AST-120 on chronic kidney disease progression in the United States of America: a post hoc subgroup analysis of randomized controlled trials. BMC Nephrol.

